# Perceptions of health care providers concerning patient and health care provider strategies to limit out-of-pocket costs for cancer care

**DOI:** 10.3747/co.v16i4.375

**Published:** 2009-08

**Authors:** M. Mathews, S. Buehler, R. West

**Affiliations:** * Division of Community Health and Humanities, Faculty of Medicine, Memorial University, St. John’s, NL

**Keywords:** Costs, financial burden, affordability, cancer care, rural

## Abstract

**Objective:**

We aimed to describe the perceptions of health care providers concerning patient and health care provider strategies to limit out-of-pocket costs for cancer care.

**Methods:**

We conducted semi-structured interviews with 21 cancer care providers (nurses, social workers, oncologists, surgeons, pharmacists, and dieticians) in Newfoundland and Labrador.

**Results:**

Patients try to minimize costs by substituting or rationing medications, choosing radical treatments, lengthening the time between follow-up appointments, choosing inpatient care, and working during treatment to minimize loss of income. Providers respond to the financial concerns of patients by helping them to access financial assistance programs, by changing chemotherapy and supportive drug prescriptions, and by shortening radiation treatment protocols. They admit patients to hospital and arrange follow-up with physicians closer to a patient’s home.

**Conclusions:**

Out-of-pocket costs resulting from cancer care are incurred at all phases of treatment and follow-up. These costs are substantial concerns for some patients and their health care providers. Encouraging communication between patients and their providers is needed to identify individuals at risk and to safely modify care plans. Tele-oncology and public drug, medical travel, and leave programs are needed to ensure that patients are better able to afford the costs related to cancer care.

## 1. INTRODUCTION

Although Canada’s universal public health insurance covers the costs of all medically necessary cancer care provided in clinical settings, patients still incur substantial out-of-pocket costs related to their care. Recent Canadian studies have shown that out-of-pocket costs for cancer care are substantial 1,2. These costs include expenses directly and indirectly related to treatment. Direct costs include drugs (prescription, alternative, and over-the-counter medicines), alternative therapies, medical supplies, home care, and nutritional supplements; indirect costs include prostheses and wigs, travel, lodging, meals, child or elder care, telephone, home support, furniture modifications, and loss of income for patients without sick leave benefits 1–7.

Out-of-pocket costs are of particular concern to low-income and rural patients. Compared with patients in higher socioeconomic groups, those in lower socioeconomic groups—such as the working poor—are more affected by the out-of-pocket burden because a greater percentage of their income will be consumed by these costs 6,8,9. To access specialized care, rural residents incur costs related to travel and lodging. These individuals are also less likely to have private health insurance that may offset out-of-pocket costs.

A number of studies have examined how patients respond to high out-of-pocket costs, but these studies have largely looked at costs related to prescription drugs and were conducted in the United States. By comparison, few studies in Canada have examined the strategies that patients and their providers use to limit out-of-pocket costs for cancer care.

We used qualitative interviews with cancer care providers to examine, from the providers’ perspective, how they and their patients respond to out-of-pocket costs. The present article is based on a larger study of out-of-pocket costs incurred by cancer patients that included both surveys of patients and qualitative interviews with care providers. Here, we report exclusively on the findings from the qualitative interviews.

## 2. METHODS

This study received ethics approval from the Human Investigations Committee of Memorial University of Newfoundland and the Newfoundland Cancer Treatment and Research Foundation Research Management Committee.

We identified potential participants from the staff list of the provincial cancer care agency. In addition, we asked participants to identify other individuals to interview. Eligible participants were actively involved in cancer care in the province, spoke English, and were willing to participate in an interview. The goal of our recruitment strategy was to interview people who would reflect a variety of opinions and experiences. The final number of interviews was determined by saturation of themes and concepts: that is, interviewing continued until no new ideas emerged.

Using a semi-structured interview guide, we generally asked each person about

- their role in the delivery of cancer care,
- how they identify financial concerns in their patients,
- the types of financial concerns that affect patient treatment decisions, and
- how those financial concerns influence treatment.

Here, we focus on the responses to the last two questions. A separate article examines responses to the second question (Mathews M, Park AD. Cancer care providers’ perceptions of barriers to identifying cancer patients in financial need. In preparation). Interviews were done in person or over the telephone and lasted up to 1 hour. Each interview was tape-recorded and transcribed verbatim. Pseudonyms were used for each informant (and are presented in this article).

We used a thematic analysis approach to analyze the transcripts. Data collected were continuously compared to data from previous interviews to identify concepts, categories, clusters, and themes 10,11. Two members of the research team independently read each transcript, identified key words, and developed a coding scheme 10,12. We coded two interviews together to develop consistent definitions of terminology and to revise and refine the coding template. We resolved disagreements in coding through consensus, creating new themes or integrating ideas within a theme where needed. The remaining transcripts were then coded using NVivo 7 software [QSR International (Americas), Cambridge, MA, U.S.A.].

We used a number of measures to enhance the credibility of our data and analysis. First, during the interviews, the interviewer conducted member-checking with the participants by summarizing responses and reporting them back to the participants to verify that that their responses had been accurately interpreted 11. By developing the coding template as a team and by independently coding and then comparing the first set of interviews, we clarified misunderstandings to make sure that the codes were consistently applied 13. We kept detailed records of the interviews (transcripts and audiotapes), field notes, and drafts of the coding template 14. Finally, we provide a thick description of the context in which we collected and analyzed the data to give readers enough detail to place the findings in similar contexts 10.

## 3. RESULTS

Between April and August 2003, we contacted 25 health care providers. All but 4 agreed to participate in an interview. Individuals who declined to participate cited busy schedules and lack of time to take part. Given the relatively small number of cancer care providers in the province, we provide minimal information on individual participants to protect their confidentiality.

Our 21 participants included nurses, social workers, oncologists, surgeons, pharmacists, and dieticians. Fifteen participants worked at the cancer care facility in St. John’s; the others worked in regional cancer clinics across the province. They had between 3 and 30 years of health care experience.

Once aware of a patient’s financial concerns, the first reaction by health care providers is to help the patient to access financial assistance, usually by referring the patient to social workers employed with the cancer treatment agency. The health care providers we interviewed noted that identifying patients in financial need is not always straightforward and that many needy patients may not be identified in a timely manner.

As summarized in Table I, health care providers also identified ways in which both they and their patients attempted to manage patient costs related to drugs and medical supplies, travel and lodging, and loss of income. Although we did not hear of a single example in which a patient refused care, participants said that financial concerns can nonetheless have a considerable impact on patients and on their care plans.

### 3.1 drugs, Medical Supplies, and equipment

Medication substitution and rationing were the most frequently cited strategies used by patients to limit out-of-pocket drug costs. As described here, patients may substitute cheaper medications for prescriptions:

We have actually had people who can’t afford some of the more expensive, newer anti-nausea drugs, and they’ve actually decided that they’re not going to take them.... They decide to go with the older, less expensive anti-nausea drugs. And [those drugs are] not always quite as effective.

Or they may ration medications to make a prescription last longer:

They know [that] if they’re going to take that painkiller every four hours, well that’s going to be six at the top of the day. If they can get away with three, you know, through a bit of suffering or what have you, then obviously, they will take half the medication.

The drugs that patients substitute or ration are “supportive” drugs (that is, to control nausea or pain) rather than chemotherapy agents and usually the ones taken outside the hospital setting. Providers generally become aware of patients rationing or substituting drugs by accident: for example, when patients do not experience the expected symptom relief, are hospitalized, or present at the emergency department for related problems.

When health care providers become aware of cost concerns, they may alter treatment protocols. For example, providers may change drug prescriptions for either chemotherapy or for supportive drugs and “try to come up with a comparable treatment [that] will be covered [by the patient’s private insurance].” Or they will ask drug companies to provide drugs at reduced or no cost: “It depends on whether ... we’ll have to get them compassionate-release drugs if they don’t have a drug plan. Sometimes it depends; we’ll change our entire chemo regimen based on geography and finances.”

Our participants suggested that patients and their providers both opt for inpatient care to limit costs related to medical equipment, drugs, and home care. Here, a nurse describes a palliative care patient who chose to die in hospital rather than incur the costs of dying at home:

She said, “No, I’m not going home; I’ll stay here.” She was 40 years old, and she was dying, and she said “No, I’m not going home. Because, if I go home, my family has to purchase the oxygen, I have to buy the antiemetics, which are $25 a pill.... No, I will stay in here, and I will use the hospital’s oxygen and I’ll take their pills.”

This nurse noted that many small communities in the province do not have a health facility in which palliative care is available; as a result, dying patients may be at a considerable distance from their friends and families.

Hospitalization was used for patients at any phase in their treatment if out-of-pocket costs or the provision of care at home posed a substantial burden:

We have had patients [who] would [receive chemotherapy] in the hospital because they can’t afford medications. We’ve admitted patients to the hospital because family members can’t take time off to look after them at home from their jobs. We certainly have admitted patients if they are not able to afford the rental of our equipment at home.

### 3.2 Travel and lodging Costs

Cancer care in Newfoundland and Labrador is largely centralized in St. John’s, the sole site at which radiation therapy is available and all the oncology specialists are located. Cancer-related surgeries are performed in the larger centres across the province, and increasingly, chemotherapy is available closer to home, either in the patient’s own community or at the closest regional centre. Patients from rural communities still face considerable travel and lodging costs, particularly to access radiation treatment. In our interviews, care providers suggested that patients may choose more radical forms of treatment in an effort to reduce travel-related costs. The most frequently cited example was of women with breast cancer choosing mastectomy over breast-conserving surgery:

So if it was their decision as to mastectomy versus lumpectomy and radiotherapy, they or I know a number of women who opted for the mastectomy because [of] the time and the finances involved and no family in [St. John’s]....

Breast conservation usually requires adjuvant radiation therapy, meaning that women have to incur the expense of undergoing radiation treatment in St. John’s. As a radiation oncologist noted, the likelihood of choosing a mastectomy was related to the proximity to the radiation treatment center: “Definitely the farther away from St. John’s, the higher the mastectomy rate.”

The health care providers in our study suggested that, in an effort to reduce the number of trips made away from home, rural residents with cancer may miss follow-up appointments or lengthen the time between follow-up visits: “They probably don’t go to the follow-up appointments that they should go to because of the expense involved, or they would choose to be followed up less frequently.”

The health care providers we interviewed also described a number of strategies that they used to limit the time during which patients would have to be away from home (and incur costs related to lodging). For example, a radiation oncologist may try to complete treatment before a weekend: “If a patient is scheduled for ... treatment on a Monday, we will usually try to double up ... treatments the week before so that they can finish on a Friday—these out of town patients.” They may also change the follow-up protocol by limiting the extent of the patient’s follow-up or by having a family physician or surgeon from the patient’s home community follow the patient. Patients are also given follow-up appointments at the regional clinics rather than in St. John’s.

A number of strategies involved coordinating or carefully scheduling appointments. For example, appointments with oncologists may be scheduled to coincide with another appointment to reduce the number of trips that patients have to make:

If they’re coming from really far away and ... surgery would be an option for treatment, then they would try and coincide the appointment visit with their surgical date so they don’t have to make the extra trip.... We will try and arrange to have investigations done in a community close to them and then [have them] arrive at the date of admission so they don’t have to go back home.

Oncology nurses also take into account travel times and road conditions when they schedule appointments:

We said, “Okay. Find out in those areas what time the taxis and the buses would get into—say, Grand Falls—from wherever they’re coming from—say, Twillingate, Gander, Bay d’Espoir, and so on,” and therefore to set up the appointment to coincide with those times. For example, if you’re going to have a clinic in Grand Falls, you would not send an appointment out to somebody in St. Alban’s for 9:00 [am].

As the last line suggests, appointment times are also carefully chosen to coordinate with bus schedules or with traveling times so that patients can avoid an overnight stay.

### 3.3 loss of income

Patients who are self-employed, seasonally employed, or employed in small business often do not have sick leave benefits and will therefore lose income for absences from work during treatment or recuperation. For some patients, the opportunity costs associated with seeking care are perceived to represent an important impediment to accessing care. For example, despite their ill health, patients continue to work during their treatment:

When you are working in a fish plant, or you’re a plant worker, you do not have any sick leave. You take a day off, that’s a day’s pay gone.... You might take a couple of days off because you’re too miserable to stand up, but then you got to go back to work if you expect to have any income for the rest of that year.

That last line reflects the seasonal nature of employment in many small communities in New-foundland and Labrador, where residents must work a minimum number of weeks to qualify for Employment Insurance if they are to have income for the remainder of the year.

Limited sick leave can also have a substantial effect on treatment protocols. Here, a health care provider describes how the radiation therapy plan can be drastically altered to accommodate the patient’s annual leave:

It’s the same as a patient coming in and saying, “I only have two weeks of annual leave. I can’t afford to take any more than that off.” ... There may be a treatment protocol that the oncologist can come up with that is pretty well the same as a longer treatment and ... if the financial part of it is weighing heavy, he may go with that.

## 4. DISCUSSION

Qualitative interviews with cancer care providers revealed various strategies perceived to be used by patients and care providers to reduce out-of-pocket costs stemming from cancer care. Although out-of-pocket costs related to drugs, medical supplies and equipment, or loss of income may affect all patients regardless where they reside, travel and lodging costs are almost exclusively borne by rural residents. Previous studies have suggested that costs related to travel can influence care decisions made by patients 5. For example, researchers in Canada have observed lower rates of breast-conserving surgery among eligible women who live in rural regions, who have longer travel times to a cancer treatment centre, or who have low income 15–18, and they have suggested that the considerable financial and social costs associated with the treatment (specifically to access radiation treatment) may discourage rural patients from choosing breast conservation.

Cost-savings strategies were identified for all phases of treatment, including surgery, radiation, chemotherapy, follow-up care, and palliative care, suggesting that each phase of care may create financial pressures. A U.S. study suggests that 18% of patients with chronic illness may underuse their medication at least once during the year 19; however, we are unable to assess the proportion of cancer patients who use cost-reduction strategies over the course of their treatment. We are currently conducting studies to identify the phases of care that pose the greatest financial burden to patients with different types of cancers.

Open communication between patients and their cancer care team is critical in identifying a safe means of reducing out-of-pocket costs for the patient. In the interviews, we noted that cancer care providers often learn that patients are rationing or substituting drugs only when the patients have poor symptom control. Elsewhere, we examined the care providers’ perceptions of barriers to identifying patients with financial concerns (Mathews M, Park AD. Cancer care providers’ perceptions of barriers to identifying cancer patients in financial need. In preparation). Consistent with other studies, we found that health care providers were very responsive to patients’ financial concerns, but that discussions of these concerns were not routine. For example, U.S. researchers report that only 15%–16% of patients ever discuss out-of-pocket drug-related costs with their physicians 12,20. Likewise, only 35% of physicians ever discussed drug costs with their patients 20. In a survey of adults with chronic illnesses, respondents gave the following reasons for not discussing drug costs with their physicians 12:

- They had not been asked (66%).
- They did not think that their health care providers could help (58%).
- They were too embarrassed (45%).
- They did not think that the issue was important (45%).
- Insufficient time was available during the visit (31%).
- There was a lack of trust (11%).

Advocates suggest that systematic screening of patients when they initially present for care may help to identify individuals at financial risk 21. However, given that the types and amounts of out-of-pocket costs may change over the course of treatment, cancer care providers should inquire about financial concerns at various phases during treatment.

System-wide initiatives are also needed to improve the affordability of cancer care. Tele-oncology programs limit travel costs by allowing patients to be seen in their home community 22,23. Catastrophic drug insurance programs and medical travel subsidies may offset costs borne by patients. The availability and terms of these programs vary by province. In addition, sickness and compassionate leave programs offered by the federal government may offset loss of income 24,25. Both of the latter programs have specific eligibility requirements and may not cover the entire period of time that an individual is unable to work.

## 5. LIMITATIONS

We conducted interviews with cancer care providers. Gathering information from patients may reveal additional strategies for limiting out-of-pocket costs and also barriers faced by patients in communicating about financial concerns with health care providers and in accessing subsidy programs. Our qualitative interviews identified strategies used by patients and providers to limit costs, but we were unable to assess how often these strategies are used. Nevertheless, a qualitative inquiry such as this study is particularly useful for generating hypotheses that can be tested using other designs and methods 11. For example, we are currently conducting studies examining how often patients use cost-saving strategies relative to actual out-of-pocket costs.

## 6. CONCLUSIONS

Out-of-pocket costs resulting from cancer care are a substantial concern for some patients and their health care providers. Patients and their care providers are both believed to use a variety of strategies to limit costs related to drugs and medical supplies, travel and lodging, and loss of income. Communication between patients and their care providers needs to be encouraged so as to identify individuals at risk and to safely modify care plans. In addition, tele-oncology and public drug, medical travel, and leave programs are needed to improve the affordability of cancer care.

## Figures and Tables

**TABLE I:** Summary of perceived patient and provider strategies to limit out-of-pocket costs for cancer care

*Type of cost*	*Strategies used by*	
	*Patients*	*Cancer care providers*
Drugs, medical supplies, and equipment	Substitute or ration medications	Change drug prescriptions
	Choose inpatient care	Admit patients to hospital
Travel and lodging	Choose radical treatment	Shorten radiation treatment
	Stretch out the time between follow-up appointments	Arrange follow-up with local surgeon or family physician
		Change appointment schedule:
		Coordinate oncologist and other appointments
		See patients at regional clinics
		Schedule appointments at convenient times
Loss of income	Limit absence from work	Shorten radiation treatment
